# Femur Fracture With Arterial Injury in an 11-Year-Old Boy: A Case Report of a Rare Association

**DOI:** 10.7759/cureus.21868

**Published:** 2022-02-03

**Authors:** Larbi Benradi, Kamal El Haissoufi, Abdellah Rezziki, Omar El Mahi, Mohamed Belahcen

**Affiliations:** 1 Pediatric Surgery, Mohammed VI University Hospital of Oujda/Faculty of Medicine, Mohammed 1st University of Oujda, Oujda, MAR; 2 Vascular Surgery, Mohammed VI University Hospital of Oujda/Faculty of Medicine, Mohammed 1st University of Oujda, Oujda, MAR

**Keywords:** child, surgery, blunt trauma, femur fracture, vascular injury

## Abstract

Post-traumatic vascular lesions of the lower extremity in children are uncommon and present some particularities in their management in comparison to those that occur in adults. Here, we report the case of an 11-year-old boy who presented with a diaphyseal fracture of the right femur associated with an injury of the homolateral superficial femoral artery after blunt trauma of the lower limb. The bone and the arterial injuries were surgically and successfully treated by an elastic stable centromedullary nailing and a venous bypass, respectively, with a good clinical and radiological evolution. The management of vascular injuries in childhood can be challenging and requires some special measures to avoid undesirable complications. Functional prognosis is generally described to be good if a rapid and adequate treatment is performed.

## Introduction

Noniatrogenic vascular injuries in children are uncommon and still not very discussed in the literature in contrast with the amount of studies already published about this topic in adults [1]. Indeed, only 0.6% of all children who presented with traumatic injuries were described to have a vascular lesion and the extremities seem more likely to be concerned [1]. Additionally and compared to adults, vascular lesions in childhood present unique characteristics including the smaller diameter of the vessels, their growing character and the great predisposition to vasospasm which may pose some challenges during their management [2,3]. In this case report, we aim to discuss the findings of an 11-year-old boy with a displaced right femoral diaphyseal fracture associated with an injury of the homolateral superficial femoral artery after blunt trauma of the extremity, and who was successfully treated in our primary academic center.

## Case presentation

**Figure 1:**
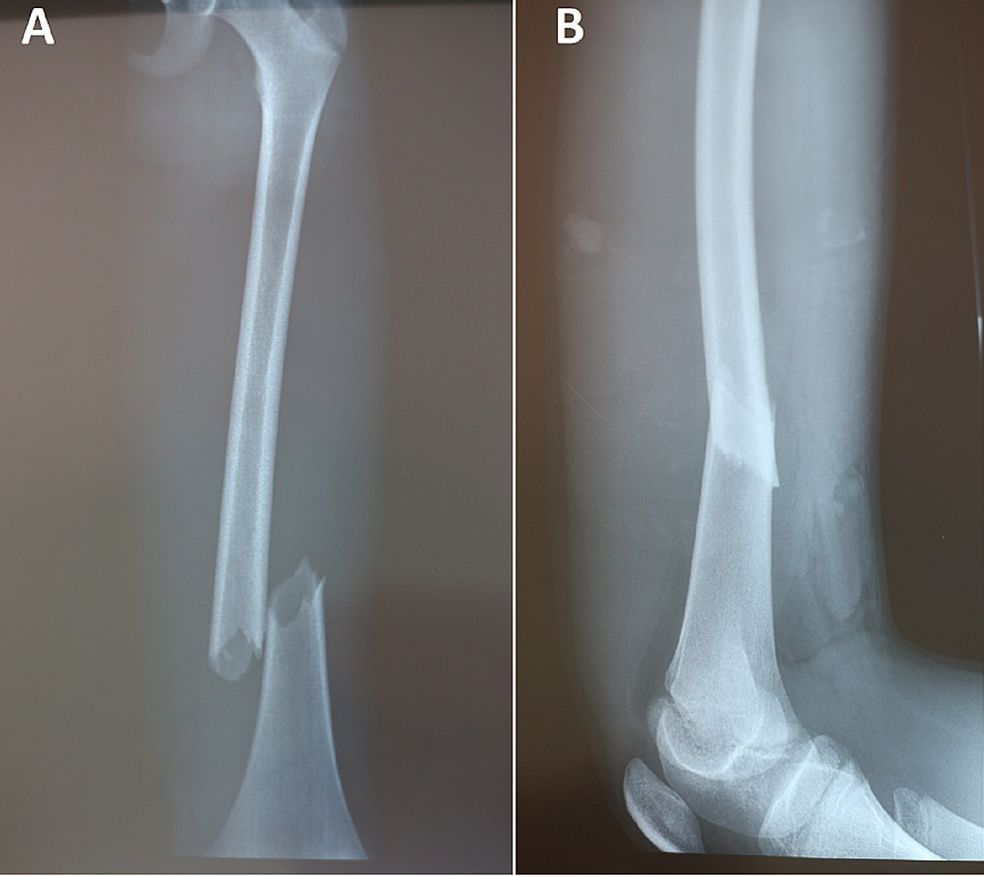
Right thigh x-ray imaging showing an overlapping and displaced distal diaphyseal fracture of the femur on anteroposterior (A) and lateral views (B)

An 11-year-old boy with no significant pathological history was admitted to the emergency department for a right lower extremity trauma after a road accident. The patient had no past surgical interventions. The clinical examination found an apyretic child and in a good general condition. A deformed right thigh with an absence of distal pulses including popliteal, posterior tibial and dorsalis pedis, coldness and paleness of the right lower limb was noted. The rest of the physical examination was without abnormalities, especially that of the spine, thorax and abdomen. X-ray imaging of the right thigh revealed an overlapping, displaced right femoral diaphyseal fracture (Figures 1A, 1B).

**Figure 2:**
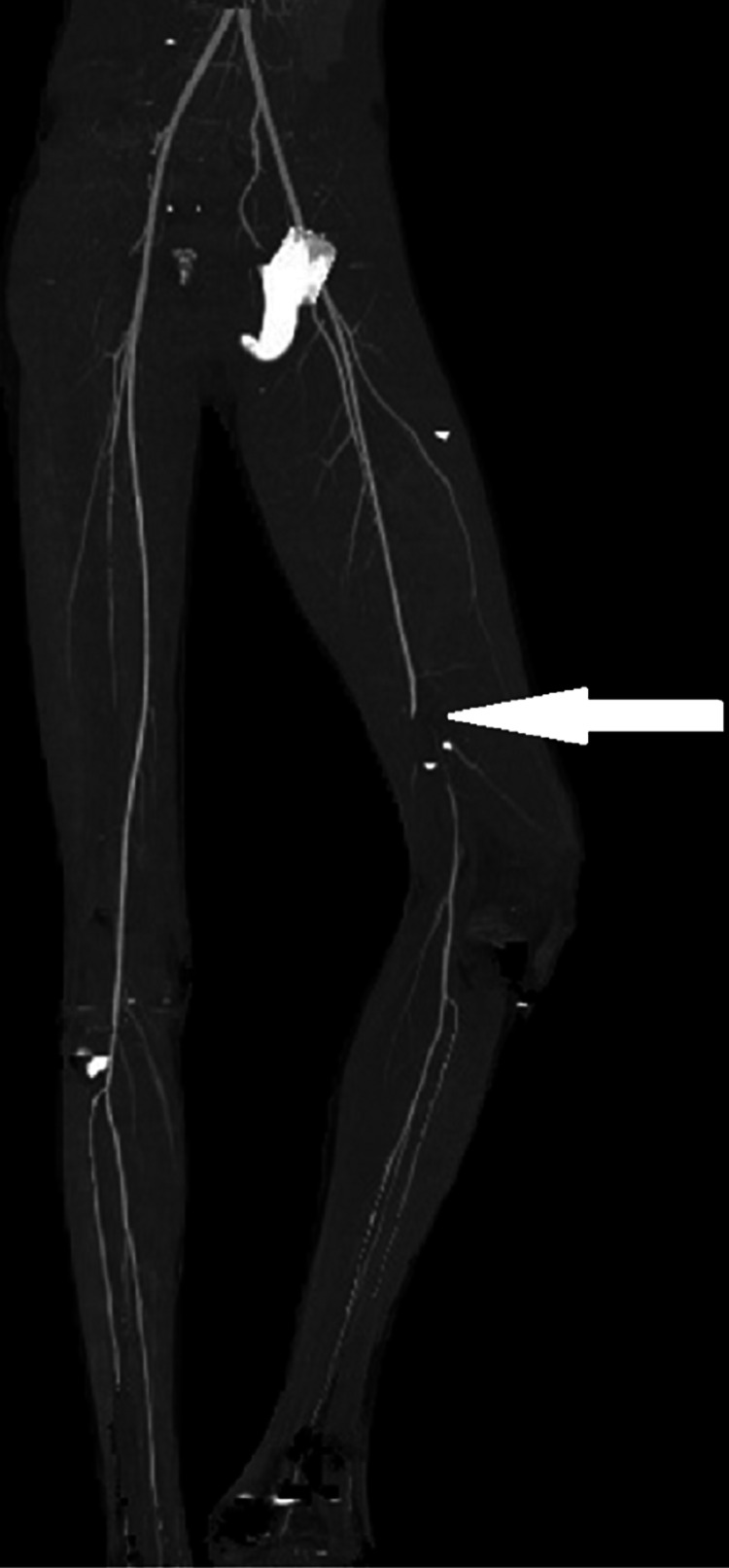
Computed tomography angiography (CTA) of the lower limb performed preoperatively showing an intimal rupture of the right superficial femoral artery with interruption of the blood flow (white arrow)

A computed tomography angiography (CTA) of the lower limb was performed and showed an intimal rupture of the superficial femoral artery with interruption of the flow at this level (Figure 2).

**Figure 3:**
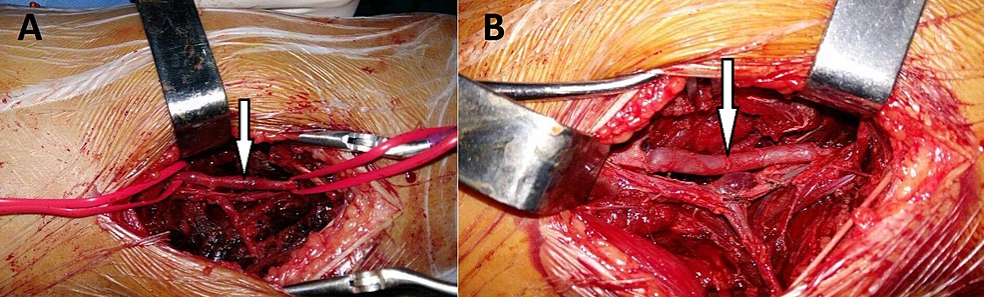
Preoperative views showing the intimal rupture of the superficial femoral artery (A) and the saphenous vein graft bypass performed (B) (white arrows)

Preoperative biological data were all within the normal range. No diagnostic challenges in our patient could be reported. The urgent therapeutic attitude was to reduce and stabilize the fracture by an elastic stable intramedullary nailing (ESIN) and the realization of a vascular bypass by saphenous vein graft (Figures 3A, 3B).

**Figure 4:**
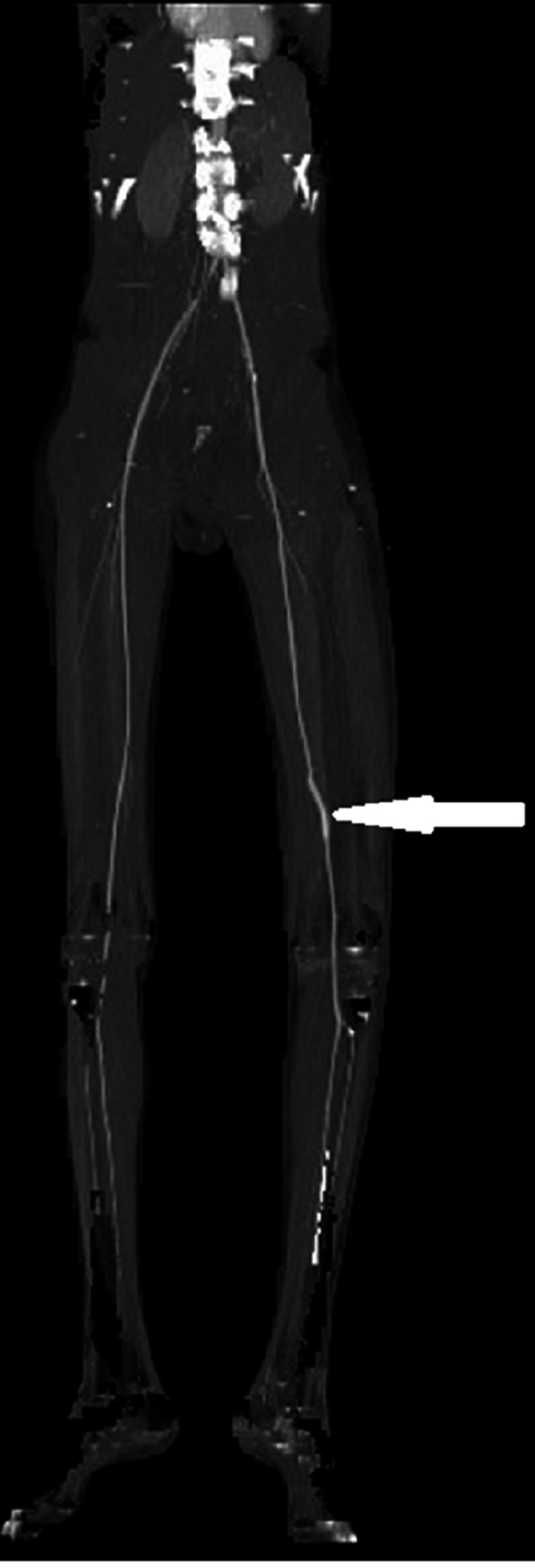
2D reconstruction of a CTA scan control performed one month postoperatively and showing a functional venous bypass (white arrow)

The postoperative time was uneventful with no hemorrhagic or infective complications. A CTA control was performed one month after and objectified a functional venous bypass (Figure 4).

The verticalization was carried out from the 30th day with partial support and total support was authorized 15 days thereafter. The child was reviewed regularly every three months for a period of 12 months. The evolution was favorable after a follow-up period of two years.

## Discussion

Trauma in patients aged less than 16 years with vascular injuries is uncommon and seen in only 0.6% to 1.4% [4,5]. Compared to adults, this incidence was reported to be significantly lower and vessels of the upper extremity are the most involved in children [4]. Within vessels of the lower extremity, injuries of a superficial femoral artery are less common than those of popliteal artery and tibial vessels and are encountered in only 3.7% of all vascular lesions in the pediatric population [4]. Penetrating mechanism is reported to be the leading cause of vascular injuries in children [6]. In our patient, blunt trauma was the mechanism of the injury of a superficial femoral artery which makes this case interesting to be reported.

Classically, vascular trauma of the lower limb must be suspected when distal pulses are absent or diminished, and if distal ischemia or hematoma is observed [7]. Remarkably, orthopedic injuries are associated with a third of cases and distal femoral fractures are the most common [7]. Obvious signs of vascular injuries are mainly enough to indicate a surgical repair and further imaging studies are required only when the lesion is questionable or when it is necessary to plan adequate surgical revascularization [7]. Moreover, conventional angiography represented the gold standard of diagnosis but it was related to significant complications in children including radiation exposure, contrast-induced nephropathy, acute femoral artery thrombosis and chronic femoral artery occlusion associated with limb growth retardation [8]. Therefore, CTA with controlled radiation exposure and minimal acceptable contrast dose was proposed as a safe and reliable alternative tool of diagnosis [8]. Similarly, our patient presented an absence of distal pulses associated with ischemia signs, and a diaphyseal femoral fracture was the only concomitant lesion. A CTA was the paraclinical radiological exam performed to plan the surgical procedure of revascularization.

A vascular repair can be avoided as long as sufficient distal perfusion of the extremity is the case [6]. Otherwise, the surgery consists of primary repair if feasible, and vascular reconstruction by autologous grafts or synthetic conduits such as polytetrafluoroethylene (PTFE) graft if not [7]. Importantly, vascular repair should be taken into consideration first before the fracture treatment when severe ischemia of the limb is the case [9]. In the present case, the fracture was firstly and rapidly stabilized by an ESIN and the vascular repair was performed by using an autogenous saphenous vein graft.

In terms of prognosis, compartment syndrome, infection, bypass thrombosis and amputation are the frequent possible complications [6]. However, limb salvage is generally the case in 95% to 97.4% of noniatrogenic vascular injuries and no death within the group with lesions of the lower extremity was reported [6,9]. Also, a significant difference concerning the fracture nonunion as well as the average time of the fracture union if an arterial lesion is associated with bone damage was reported [10]. Therefore, inadequate blood circulation can compromise the normal growth of affected bones, resulting in limb length differences and long-term disability. However, long-term functional results were described to be good with a return to normal activity in most of the cases for patients who underwent bypass surgery [9]. If not, an adequate functional return was the case for the rest of the patients despite associated nerve injuries [9]. For our patient, the fracture healing time was without abnormalities with a functional venous bypass, and a total return to the normal function of the limb was obtained during a postoperative follow-up period of two years.

## Conclusions

Post-traumatic vascular injuries, especially after blunt trauma of the extremity, are rarely seen in children. Their management can be challenging and requires some particularities since pediatric vessels present unique characteristics. The overall prognosis is described to be good if this management is attempted by experienced surgeons.
